# Evaluation of the Effects of Undenatured Type II Collagen (UC-II) as Compared to Robenacoxib on the Mobility Impairment Induced by Osteoarthritis in Dogs

**DOI:** 10.3390/vetsci6030072

**Published:** 2019-09-04

**Authors:** Marzia Stabile, Rossella Samarelli, Paolo Trerotoli, Laura Fracassi, Luca Lacitignola, Antonio Crovace, Francesco Staffieri

**Affiliations:** 1 Department of Emergency and Organ Transplantation, PhD in Tissues and Organs Transplantation and Cellular Therapies, University of Bari, 70010 Bari, Italy; 2 Department of Emergency and Organ Transplantation, Section of Veterinary Clinics and Animal Production, University of Bari, 70010 Bari, Italy; 3 Department of Biomedical Sciences and Human Oncology, University of Bari, 70010 Bari, Italy

**Keywords:** osteoarthritis in dogs, chronic disease, management of pain, multimodal analgesia

## Abstract

Osteoarthritis (OA) is a chronic disease that requires a multimodal therapeutic approach. The aim of this study was to evaluate the effects of undenatured type II collagen (UC-II) as compared to robenacoxib in dogs affected by OA. Our hypothesis was that the two compounds would be similar (non-inferiority) in improving mobility. To test this hypothesis, a complete orthopedic examination, x-ray and the Liverpool Osteoarthritis in Dogs (LOAD) survey were performed in dogs affected by OA before and after the treatments. The study was designed as a clinical, randomized, controlled and prospective study. Sixty client-owned dogs were randomized in the R group (*n* = 30, robenacoxib 1 mg/kg/day for 30 days) and in the UC-II group (*n* = 30, UC-II 1 tablet/day for 30 days). Thirty days after the beginning of the treatment (T30), the dogs were reassessed for the LOAD, MOBILITY and CLINICAL scores. Based on the data obtained from the study, a significant reduction in LOAD and MOBILITY scores was recorded between T0 and T30 with a similar magnitude among the two groups (R = 31.5%, *p* < 0.001; UC-II = 32.7%, *p* = 0.013). The results of this study showed that UC-II and robenacoxib were able to similarly improve mobility of dogs affected by OA.

## 1. Introduction

Osteoarthritis (OA) is a chronic inflammatory disease that affects the entire joint tissue [1]. The new evidences related to the pathogenesis of this disease consider it a multifactorial disorder in which low-grade chronic inflammation has a central role. This inflammation plays a early stage role in the course of OA, as a result of interactions between the immune system and multiple factors including local tissue damage and metabolic dysfunction [2]. Osteoarthritis is also considered a functional disorder with subsequent alteration of mechanical properties resulting in decreased stability, movement and loading.

It was estimated that 20% of the canine population over one year of age is affected by OA and the most involved joints are the hip, elbow and stifle [3,4]. Subjects suffering with OA tend to be less active, have difficulty of movement, cracking joints, stiffness, muscle wastage and visible pain [5]. The combination of history, orthopedic examination and radiographic evaluation are the main clinical steps for the diagnosis of OA in canine patients [1,5].

Additionally, the use of the clinical metrology instruments (CMIs) allow to collect clinical data based on the owners’ observations [6]. Liverpool Osteoarthritis in Dogs (LOAD) is an owner-completed survey, subjective, a validated CMI, easy to use, with a demonstrated correlation with the force platform. The instrument assesses locomotor function with a series of 13 multiple-choice questions with descriptive answers. These are given individual numeric ratings (0–4) and added to achieve an aggregate score (0–52) that reflects the degree to which mobility is impaired [7,8].

The management of OA involves various pharmacological and non-pharmacological treatments, in a “multimodal” approach, including the relieve of pain by reducing the inflammation and slowing the progression of the cartilage damage, to improve the patient’s quality of life [4,8,9].

Non-steroidal anti-inflammatory drugs (NSAIDs) are the cornerstone of pharmaceutical therapy for OA thanks to the ability to reduce the inflammation and treat pain [4,5]. However, the chronic use of NSAIDs may be hampered by the development of gastrointestinal and/or renal side effects [10,11,12,13].

Robenacoxib belongs to the new generation of NSAIDS characterized by a high selectivity for the cyclo-oxygenase (COX)-2 enzyme (COXIB) [14,15]. It is a drug developed solely for companion animal use with several properties of interest for use in dogs: it has a fast onset and the availability of both injectable and oral formulations; it is cleared from the central body compartment, but persists at the site of inflammation (tissue selectivity) [16,17] and it has less side effects than others non-selective NSAIDs [18]. In the European Union, robenacoxib is registered for the treatment of pain and inflammation associated with OA and surgery in dogs, showing safety also for long-term use, at the dosage of 1–2 mg/kg [18,19].

Recently, it has been demonstrated that undenatured type-II collagen (UC-II) from a chicken sternum markedly reduced inflammation and pain during OA in experimental animals and dogs, improving their quality of life [3,20]. UC-II has been shown to act on joint inflammation by interfering with the local immunity: small amounts of undenatured type-II collagen (10 mg active UC-II), orally administered, inhibits the immune response targeting type-II collagen in joint cartilage. This mechanism is known as oral tolerance. Adverse effects have not been noticed in humans, dogs or horses [17,19]. Up to date the efficacy of UC-II has been proven as compared with glucosamine and chondroitin in dogs with OA [3]. For this reason, UC-II appears as a valid diet supplement for the management of OA in combination with NSAIDs.

Our hypothesis was that UC-II may provide an improvement in the mobility and clinical signs associated with osteoarthritis non-inferior to that produced by robenacoxib. The aim of this study was to determine if UC-II is non-inferior to robenacoxib for the management of dogs affected by lameness induced by OA. The primary outcome was the improvement of mobility impairment. To test the hypothesis a complete orthopedic examination and the LOAD survey were performed in dogs affected by OA before and 30 days after the beginning of the treatments.

## 2. Materials and Methods

### 2.1. Study Protocol

The study was designed as a clinical, randomized, controlled and prospective study, and it was approved by the ethical committee of the Department of Emergency and Organ Transplantation of the University of Bari, Italy (approval # DETO/223/III/13).

Client-owned dogs affected by OA, conducted at the Surgical Unit of the Section of Veterinary Clinics and Animal Production of the Department of Emergency and Organ Transplantation of the University of Bari, were enrolled.

All patients underwent a complete physical examination, clinical chemistry and hematological evaluation at the time of presentation.

The inclusion criteria were: history of mobility impairment as referred by the owner, presence of lameness, pain and radiographic signs of OA (articular incongruence, subchondral sclerosis, presence of osteophytes and bone deformation) in at least one appendicular joint based on the veterinarian evaluation and at least 3 weeks of washout period from any previous anti-inflammatory/analgesic/nutraceutical therapy. The exclusion criteria were: the diagnosis of neurological disease, the presence of comorbidity such as major cardiovascular and respiratory dysfunctions and other chronic affections (dental, renal, hepatic diseases) as well as the cases in which no signs of OA were evident at the radiographic examination.

All the dogs included in the study underwent a complete physical examination which allowed to define the characteristics of the lameness based on the patient’s history and the veterinarian observation (gait evaluation) and was scored as follows: 1 = no lameness; 2 = temporary mild/moderate (weight-bearing) lameness; 3 = persistent mild/moderate lameness with episodes of acute severe (non-weight-bearing) lameness; 4 = severe and persistent lameness and/or with the involvement of more than one joint. Only dogs characterized by a lameness score of 2, 3 and 4 were included in the study.

The orthopedic examination included the following evaluations: posture, mobility, pain at the manipulation of the affected joint and assessment of the range of motion (ROM). Each evaluation was scored from 1 to 4 as follows: 1 = pre-clinical; 2 = mildly abnormal; 3 = moderately abnormal; 4 = severely abnormal. The highest grade recorded among the parameters assessed defined the severity of the OA in each dog and was indicated as the CLINICAL score.

At the time of the clinical examination, owners were asked to complete the Liverpool Osteoarthritis in Dogs (LOAD) [7] survey and based on the score (LOAD score) each patient was assigned to a specific grade of mobility alteration (MOBILITY score) [8]: 1 = mild (<11); 2 = moderate (11–20); 3 = severe (21–30); 4 = extreme (31–52) [8].

The osteoarthritic joints were further evaluated with a radiographic examination and were scored based on the following scheme (X-RAY score): 1 = at risk (radiographic evidence of predisposing factors such as articular incongruence); 2 = mild (evidence of articular incongruence, possible subchondral sclerosis, absence of osteophytes); 3 = moderate (evidence of articular incongruence, evident subchondral sclerosis, scarce osteophytes); 4 = severe (evidence of articular incongruence, evidence of subchondral sclerosis, presence of osteophytes, bone deformations).

At the time of OA diagnosis (T0), all the patients were randomly (excel-based system) assigned to 2 groups that received a different pharmacological treatment: Group R = robenacoxib (Onsior^®^) 1 mg/kg/day orally (OS) for 30 days [18,19] and group UCII = UC-II (Flexadin^®^Advanced) 1 tablet (40 mg) per day OS for 30 days [3,20,21,22]. Owners were instructed to give the dose at approximately the same time each day, without food and at least at one hour from the next meal. Moreover, a total of 20 minutes’ walk during the day was suggested to promote the mobility of the patient. No modification of the diet was suggested for the study time [23].

The clinician that performed the clinical examination and assigned the specific scores was unaware of the treatment plan, which was managed by an operator (MS) not involved in the clinical examinations of the dogs.

Thirty days after the beginning of the treatment (T30), dogs were reassessed for the MOBILITY and CLINICAL scores.

Dogs were excluded from the study and were treated based on the judgment of the clinician in charge, if during the treatment they showed adverse reactions and/or required additional/alternative drugs due to the worsening of the clinical conditions.

### 2.2. Statistics Analysis

A power calculation on the primary efficacy variable LOAD score for the non-inferiority approach was employed to test the efficacy of the UC-II compared to robenacoxib, (R versus UC-II) for the management of osteoarthritis. [24] According to the European Medicines Agency guidelines [25], the treatment difference between the reference drug (robenacoxib) and the test compound (UC-II) should remain above the lower limit of clinically relevant differences. The clinically acceptable limit, or margin (δ), has been considered as a 20% variation from the reference mean of the LOAD score, that in some preliminary cases resulted in 6.7 with a standard deviation of 2. The null hypothesis of non-inferiority would be rejected, with a type I error of 5%, if the one sided 95% confidence interval lower bound is above the a priori lower margin (δ = −1.34) of the reference group. The sample size was 28 for each group. (Sealed Envelope Ltd. London, 2012. Power calculator for continuous outcome non-inferiority trial. https://www.sealedenvelope.com/power/continuous-noninferior/).

The primary variable, LOAD score, approached the normal distribution and results are shown as mean ± standard deviation. The analysis was performed with a repeated measures ANOVA model to evaluate difference between groups, between time visits and interactions. To evaluate non-inferiority, the one sided 95% confidence interval of the difference in the LOAD score between treatments at T30 and of the difference between the improvement of the LOAD score of the two drugs was determined.

The CLINICAL score, the X-RAY score and the MOBILITY score have been treated as categorical variables with levels that goes from 1 to 4 corresponding to an increasing level of severity. Comparisons of percentages of different levels of severity between treatment were performed by chi-square test for independent samples. The CLINICAL score was evaluated at two different time points, therefore the analysis consisted of defining a new categorical variable with these possible results that summarize changes from the first visit to the last check: still score 4 or 3, still score 1, still score 2, from any score to score 1 and from any score to score 2. Percentages between treatments were compared by a Fisher exact test and *p*-values were adjusted for multiple comparisons. Qualitative variables were summarized as count and percentages and comparisons between independent groups were performed by chi-square tests. Data have been analyzed by Med.Calc 14.2 and SAS 9.4 software for personal computers.

## 3. Results

**Figure 1:**
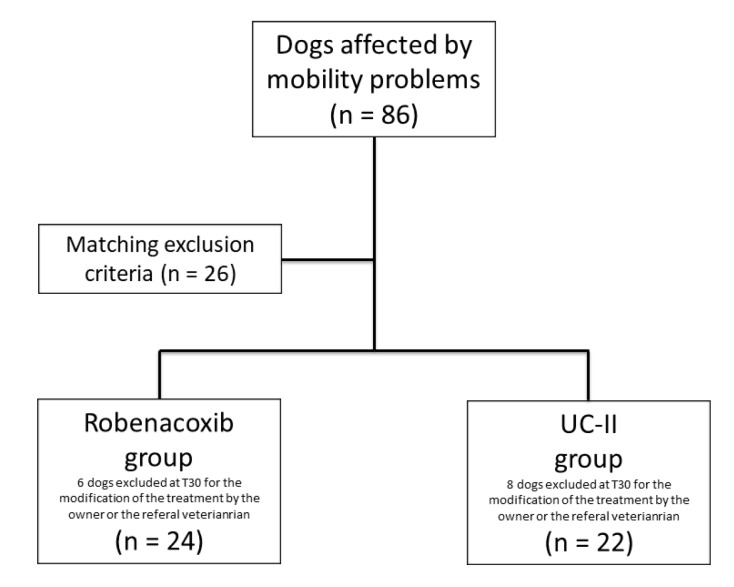
Flow chart concerning the population of dogs included in the study.

At the first consultation and clinical screening the subjects with mobility problems were 86 and 26 dogs have been excluded because of matched exclusion criteria. Sixty subjects were included in the study: 30 in the R group and 30 in the UC-II group (Figure 1). In six dogs of the R group and eight of the UC-II group, a follow-up was not available because the owner or the referral veterinarian decided to discontinue or modify the treatment. In none of these cases adverse effects were reported to us. A total of 46 dogs completed the study: 24 belonging to the R group and 22 to UC-II group.

The breeds of dogs that completed the study included: Mixed breed (8), German Shepherd (7), Rottweiler (4), Cane Corso (4), Labrador Retriever (4), Golden Retriever (3), Dogo Argentino (2), Turkemen Shepherd (2), Maremmano-Abruzzese Sheepdog (2), Beagle (2), American Staffordshire (2), Pitt Bull, Segugio Italiano, Neapolitan mastiff, Newfoundland, Boxer and St. Bernardo. None of the dogs included in the study experienced complications or adverse reactions as reported by the owner.

In the R group (*n* = 24) the subjects were 12 females (5 neutered) and 12 males (3 neutered) (age = 4.7 ± 3.7 years, weight = 26.4 ± 12.8 kg) and in the UC-II group (n = 22) the subjects were 10 females (6 neutered) and 12 males (3 neutered) (age = 5.0 ± 3.4 years, weight = 32.1 ± 11.4 kg). There were no significant differences in body weight, age and sex between the two groups.

Among the entire population the most affected joint was the hip (30.4%) followed by the elbow (26.1%), stifle (19.6%) and shoulder (2.2%). In 21.7% of the cases dogs were affected at multiple joints. Distribution of the affected joints was similar between the two groups. Particularly, in the R group the hip, elbow, stifle and multiple joints were affected in equal percentages (25%) and in none of the dogs the shoulder was affected. In the UC-II group the most affected joint was the hip (36.4%), followed by the elbow (27.3%), multiple joints (18.2%), stifle (13.6%) and in only one patient the shoulder was affected (4.5%).

The data obtained by the clinical evaluation (and recorded with the CLINICAL score) showed that 37% of the entire population was moderately (clinical score = 3), 30.4% mildly (score 2) and 28.3% severely (score 4) affected. A total of 4.3% of dogs were pre-clinical (score 1).

The MOBILITY score showed that 45.7% of subjects had severe mobility impairment, 26.1% moderate, 13% severe and 15.2% mild.

The X-RAY score showed that the 37% of the entire population had mild radiographic alterations, followed by 32.6% with moderate modifications and 17.4% with severe modifications. The 13% of subjects were in the “at risk” category.

Data relative to the CLINICAL, MOBILITY and X-RAY scores at the time of the first evaluation, divided for the treatment groups, are reported in Table 1.

Table 2 reports the changes in CLINICAL score from T0 to T30 by treatment groups; the percentages of subjects with a high score and stayed at the same score (still score 4 or 3) was higher in the UC-II group as compared to the R group and the difference was statistically significant after being adjusted for multiple comparisons (*p* = 0.039). Other results were not statistically significant between treatment groups.

**Figure 2:**
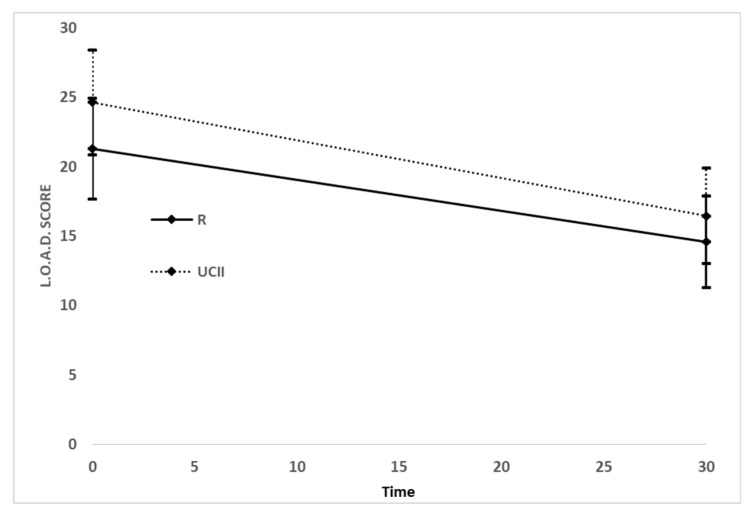
Means and their confidence intervals at 95% of the Liverpool Osteoarthritis in Dogs (LOAD) score by treatment group, at each time of the study.

Based on the data obtained from the owner evaluation, a significant reduction in LOAD and MOBILITY scores was recorded between T0 and T30 with a similar magnitude among the two groups (Figure 2): R changes from T0 21.29 (95% CI 18.52 to 24.06) to T30 14.58 (95% CI 11.65 to 17.51) and UC-II changes from T0 24.63 (95% CI 19.84 to 29.42) to T30 16.45 (95% CI 12.47 to 19.88). There was not a statistically significant difference between treatments (F = 1.27, *p* = 0.2665), nor for the interaction time and treatment (F = 0.67, *p* = 0.4163). There was, instead, a significant effect for time (F = 68.77, *p* < 0.0001). Briefly, both drugs were effective on the LOAD score, that decreased from T0 to T30 in both groups.

**Figure 3:**
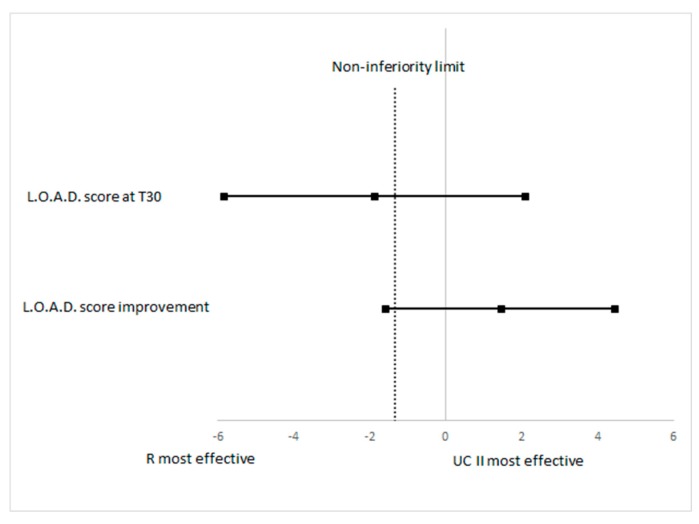
Plot of the 90% confidence limits of the difference in LOAD score at T30 and of improvement in LOAD score between R and UCII groups. The vertical dashed line is the non-inferiority limit.

The evaluation of non-inferiority looking at the LOAD score at T30 has shown a difference between treatment −1.87 and its one-sided non-inferiority limit was −5.8275, which is lower than the planned limit (Figure 3). To evaluate non-inferiority on the primary outcome measure the improvement of the LOAD score between groups was determined (R vs. UC-II): the mean LOAD decrease was −6.71 (95% CI −8.71 to −4.71) for R and it was −8.18 (95% CI −11.41 to −4.96) for UC-II. The difference in R vs UC-II was 1.47 and it was not statistically significant between the treatments (*p* = 0.4163). The one-sided lower 95% confidence limit for the non-inferiority was −1.57, which is lower than the pre-defined non-inferiority of −1.34, therefore results should be considered as inconclusive (Figure 3) for the non-inferiority evaluation.

## 4. Discussion

The results of this study show that UC-II, similarly to robenacoxib, is able to improve the mobility impairment, as assessed by the LOAD of dogs affected by a mild/moderate lameness induced by OA. Regarding the CLINICAL score (posture, mobility, pain at manipulation and ROM), despite both treatments improving the score, UC-II was not as effective as robenacoxib in most severe cases (scores 4 and 3). Accordingly, the hypothesis of non-inferiority between the two compounds was refuted.

Acute, subacute or chronic injuries, often in the context of other risk factors, can trigger a progressive cycle of local tissue damage, failed tissue repair and inflammation, resulting in further cartilage loss and progressive joint degeneration over time [26,27]. These events trigger an immune response that results in a chronic, low-grade inflammation and, ultimately, development of clinical OA [27,28]. In this context, type II collagen is among the molecular components and mechanisms that could transduce joint trauma, chronic injury or overuse into inflammatory processes.

The efficacy of NSAIDs to treat the OA symptoms is well known and this class of drug should be considered the gold standard treatment for OA. Robenacoxib is an NSAID, selective for the COX-2, which proved to be effective in treatment of OA in dogs [29]. Our results confirmed that robenacoxib was effective in the improvement of the mobility impairment induced by naturally occurring OA in dogs. Similarly, another study proved that oral robenacoxib (1 mg/kg, once daily), administered for 28 days, decreased the lameness scores and improved the radiographic scores of arthritic joints [29]. Moreover, the levels of c-reactive protein (CRP), which is an acute phase protein and highly sensitive indicator of inflammation in the synovial fluid, decreased significantly after 28 days of treatment [29]. The same study also proved that robenacoxib produce persistent inhibition of PGE2 in the inflammatory exudate despite rapid clearance from plasma (tissue selectivity) [29].

In our study robenacoxib was used as a method of comparison (reference drug) for the effects of UC-II, which showed to be effective in improving the mobility impairment as assessed by the LOAD questionnaire and thus based on the activities of the dog evaluated during the regular day life by the owner. LOAD has been validated for use in assessment of locomotor function in wider OA where a weak correlation with objective kinetic data and significant moderate correlations between LOAD and other CMIs (Helsinki Chronic Pain Index and the Canine Brief Pain Inventory) have been shown [7,8]. LOAD has also been used in assessment of long-term functional outcome following total hip replacement in dogs and a minimum reduction of 20% of the score proved to be of clinical impact [30].

Regarding the CLINICAL score, the two groups were inhomogeneous at the first evaluation, with the R group showing more severe clinical signs compare to UC-II group. This limitation could have affected the consistency of the results of the study in terms of improvement of the clinical signs. Nevertheless, dogs with more severe CLINICAL scores (4 and 3) have been more responsive to robenacoxib than UC-II, while the two treatments were similarly effective in mild and moderate cases. In the UC-II group the incidence of hip OA was higher (36.4%) than the R group (25%), a condition that could have influenced the efficacy of the treatment due to the reduced responsiveness of this specific site [31]. Nevertheless, these results indicate that despite UC-II being effective in treating mild/moderate clinical signs of OA, in more severe cases NSAIDs should be considered the first treatment option. The CLINICAL score proposed in this study was used as a way to quantify the orthopedic clinical examination of the dogs, but its clinical impact was not determined and is unknown. Therefore, it would be interesting to consider this aspect in the future and larger clinical studies.

In previous studies it was demonstrated that UC-II, in humans [21], dogs [3,20] and horses [22], shows a reduction of pain and inflammation in cases of OA. UC-II accomplishes its function through a process known as “oral tolerance”, starting in the small intestine with the oral daily intake of a small amount of type II collagen [32]. Accordingly, a T-cell response is activated at the level of the Gut-Associated-Lymphoid-Tissue (GALT), in the Peyer’s patches. After this stimulation, regulatory T helper 2 and 3 (Th2 an Th3) cells migrate from the GALT through the lymphatic system into the peripheral circulation and, when they match the type 2 collagen as an antigen, secrete cytokines (ex. TGF-b, IL-4, IL-10) that results in the downregulation of the Th1 cells, which are involved in producing the inflammation and destruction of collagen in OA.

In a previous study in arthritic dogs, Gupta et al. [3] evaluated the efficacy and safety of UC-II alone or in combination with glucosamine and chondroitin in terms of the reduction of pain by ground force plate. They demonstrated how dogs in the UC-II group showed significant reduction of pain by day 60, with a maximum reduction observed on day 150 and the peak vertical force were significantly increased, since the 90th day of treatment, compared with the others groups, indicating a decrease in arthritic-associated pain. In our study the clinical assessment was performed at 30 days and it showed a significative improvement in both the CLINICAL and MOBILITY (LOAD) scores, proving that this treatment can be effective already after one month of administration. Nevertheless, the oral tolerance is a mechanism that need at least 2–3 weeks to start to be effective but can take up to three months to be fully activated [32,33]. Thus, we can suppose that the effects of UC-II could have been even more important after a prolonged time of administration. However, the slow onset time of the clinical effects of the UC-II cannot justify its use as the first choice when an immediate anti-inflammatory effect is required. On the other hand, UC-II should not have the typical side effects of the NSAIDs and thus it could be used as long-term treatment. In the opinion of the authors the best collocation of UC-II in the multimodal management of OA could be the long-term use to support and continue the effects of the NSAIDs.

The data obtained from this study rejected our hypothesis of non-inferiority between the two treatments. However, we should consider that despite the number of dogs included in the two groups being adequate based on the sample size calculation, six subjects in the R group and eight dogs in the UC-II groups have not completed the trial and thus from this point-of-view the study should be considered underpowered. The reasons for the discontinuation of the study were not related to the occurrence of side effects but rather to the modification of the therapy based on the owner and/or referral veterinarian decision. Another important factor that may have influenced the evaluation of the non-inferiority is the inhomogeneity of the two groups at the first examination.

The absence of a negative control group should be considered another important limitation of the study. Indeed, it has been proved that the placebo effect in this type of study can occur from the caregiver and the veterinarian [34]. Thus, these results should be carefully interpreted in terms of clinical efficacy of UC-II, which will require further prospective clinical, blinded studies to confirm our results. Moreover, we should also consider the fact that the owners were not blinded to the treatment and this could have affected in some way the outcome of the LOAD questionnaire. Indeed, Jaeger et al. [35] demonstrated that, in studies in which the outcome is based on the client evaluation, the owner that knows their animal is on a standard treatment (in our case robenacoxib) may react unfavorably if they are made aware that other patients are receiving a new therapy (UC-II in the current study).

## 5. Conclusions

Undenatured type 2 collagen, after 30 days of administration, improves the mobility of dogs affected by OA on a magnitude similar to robenacoxib. UC-II is more effective as a singular therapy in mild and moderate cases of OA, based on the clinical and mobility evaluations. These results open new therapeutic windows in the treatment of OA in dogs and, in particular, if confirmed by future studies, UC-II can be a valid support in the multimodal approach to OA treatment. Considering its innovative mechanism of action, it could be used in association or as an alternative to NSAIDs administration depending on the clinical severity of the OA.

## Figures and Tables

**Table 1:** CLINICAL, MOBILITY and X-ray scores of the dogs included in the study at the first evaluation. 1 = preclinical; 2 = mildly abnormal; 3 = moderately abnormal; 4 = severely abnormal. The Table report the number (*n*) of dogs and the percentage (%) compared within the treatment group. R = robenacoxib group; UCII = undenatured type 2 collagen.

Parameters	GROUP	SCORE				*p*-Value
		1	2	3	4	
**CLINICAL SCORE *n* (%)**	*R*	1 (4.6%)	10 (41.6%)	11 (45.8%)	2 (8.3%)	0.0169
	*UCII*	1 (4.5%)	4 (18.2%)	6 (27.3%)	11 (50.1%)	
**MOBILITY *n* (%)**	*R*	3 (12.5%)	6 (25.0%)	15 (62.5%)	0 (0.0%)	0.0192
	*UCII*	4 (18.2%)	6 (27.3%)	6 (27.3%)	6 (27.3%)	
**X-ray *n* (%)**	*R*	3 (12.5%)	9 (37.5%)	8 (33.3%)	4 (16.7%)	0.998
	*UCII*	3 (13.6%)	8 (36.4%)	7 (31.8%)	4 (18.2%)	

**Table 2:** Count and percentages of changes in the CLINICAL score recorded in the two groups of treatment from the beginning of treatment (T0) and after 30 days (T30). R = robenacoxib 1 mg/kg/day for 30 days; UC-II = undenatured type II collagen 1 tablet (40 mg)/day for 30 days.

	ROBENACOXIB		UC-II		*p*-Value	
	n	%	n	%	Raw	Adjusted
Still score 4 or 3	4	16.7%	11	50.0%	0.0175	0.039
From any score to score 2	7	29.2%	6	27.3%	0.6797	1
From any score to score 1	7	29.2%	2	9.1%	0.9841	1
Still score 2	5	20.8%	2	9.1%	0.9382	1
Still score 1	1	4.2%	1	4.5%	0.7333	1
Total	24	100.0%	22	100.0%		
